# Provider and User Acceptability of Integrated Treatment for the Control of Malaria and Helminths in Saraya, South-Eastern Senegal

**DOI:** 10.4269/ajtmh.23-0113

**Published:** 2023-09-18

**Authors:** Muhammed O. Afolabi, Aminata Diaw, El Hadji Babacar Fall, Fatimata Bintou Sall, Adams Diédhiou, Amadou Seck, Baba Camara, Diatou Niang, Isaac A. Manga, Ibrahima Mbaye, Ndèye Mareme Sougou, Doudou Sow, Brian Greenwood, Jean Louis A. Ndiaye

**Affiliations:** ^1^ Department of Disease Control, London School of Hygiene & Tropical Medicine, London, United Kingdom;; ^2^ Faculté de Medecine Pharmacie d’Odonto-Stomatologie, Université Cheikh Anta Diop, Dakar, Senegal;; ^3^ Service de Parasitologie et Mycologie, Université de Thies, Thies, Senegal;; ^4^ Hospital Administration, Saraya Health Centre, Saraya, Senegal;; ^5^ Service de Parasitologie et Mycologie, Université Gaston Berger de Saint-Louis, Saint-Louis, Senegal

## Abstract

Integration of vertical programs for the control of malaria, schistosomiasis, and soil-transmitted helminthiasis has been recommended to achieve elimination of malaria and neglected tropical diseases (NTD) by 2030. This qualitative study was conducted within the context of a randomized controlled trial to explore the perceptions and views of parents/caregivers of at-risk children and healthcare providers to determine their acceptability of the integrated malaria-helminth treatment approach. Randomly selected parents/caregivers of children enrolled in the trial, healthcare providers, trial staff, malaria, and NTD program managers were interviewed using purpose-designed topic guides. Transcripts obtained from the interviews were coded and common themes identified using content analysis were triangulated. Fifty-seven study participants comprising 26 parents/caregivers, 10 study children aged ≥ 10 years, 15 trial staff, four healthcare providers, and two managers from the Senegal Ministry of Health were interviewed. Thirty-eight of the participants (66.7%) were males, and their ages ranged from 10 to 65 years. Overall, the integrated malaria–helminth treatment approach was considered acceptable, but the study participants expressed concerns about the taste, smell, and side effects associated with amodiaquine and praziquantel in the combination package. Reluctance to accept the medications was also observed among children aged 10 to 14 years due to peer influence and gender-sensitive cultural beliefs. Addressing concerns about the taste and smell of amodiaquine and praziquantel is needed to optimize the uptake of the integrated treatment program. Also, culturally appropriate strategies need to be put in place to cater for the inclusion of children aged 10 to 14 years in this approach.

## INTRODUCTION

Coinfection with malaria and helminths including soil-transmitted helminths (STH) and schistosomiasis are prevalent among preschool and school-age children living in sub-Saharan Africa (SSA), where environmental and host factors contribute to the coexistence of the multiple infections. Malaria and helminth coinfection is associated with anemia, poor physical and cognitive development, and increased mortality.^1^^–^^3^ Current vertical programs recommended by the WHO for the control of malaria and helminths among children have achieved appreciable reductions in the morbidity and mortality associated with the infections. For example, Seasonal Malaria Chemoprevention (SMC), recommended by the WHO in 2012 to prevent malaria infections during high transmission seasons, has provided high protective efficacy against malaria and prevented many malaria cases and deaths.^4^^,^^5^ Seasonal Malaria Chemoprevention is implemented through monthly administration of sulfadoxine-pyrimethamine plus amodiaquine (SP+AQ) to children aged 3 to 59 months living in the Sahel and sub-Sahel regions of Africa, usually through house-to-house visits during the months of the peak malaria transmission season. The remarkable success of SMC has led to the recent WHO recommendation of its extension to other at-risk age groups and highly seasonal malaria transmission settings outside the Sahel region.^6^

Similarly, WHO recommends integrated approaches to the control and elimination of schistosomiasis and STH through preventive chemotherapy (PC), which comprises periodic administration of anthelminthic medicines (praziquantel for schistosomiasis and albendazole or mebendazole for STH). However, a WHO report on endemic countries across SSAcshowed a substantial reduction in PC coverage for preschool-age children from a coverage of 53.3 million in 2019 to barely 7 million in 2020. In the same year, nearly 71 million school-age children (44.1% coverage) were treated for STH, and approximately 55 million were treated for schistosomiasis (46.6% coverage). This development contributed to the launch of a new neglected tropical diseases (NTD) road map target that reinforced the importance of the paradigm shift from control to elimination of schistosomiasis and STH as public health problems in all endemic countries by 2030.^7^^,^^8^

The overlap in the epidemiology of malaria and helminths has been identified as a potential area to exploit for the development of an integrated control strategy that could help to achieve the WHO targets of eliminating malaria and helminths by 2030.^3^^,^^9^ To our knowledge, no published studies have assessed the feasibility and effectiveness of combining mass drug administration (MDA) for schistosomiasis and STH with SMC, despite the overlap in target populations and potential distribution mechanisms. Furthermore, the few studies^10^^–^^13^ that evaluated the impact of adding anthelminthic drugs to antimalarial medications among school children did not report the acceptability of this approach. Acceptability becomes a crucial issue because of the potential pill burden and the potential of increased adverse drug reactions resulting from co-administration of anthelminthic and SMC drugs. The bitter taste of amodiaquine and praziquantel is known to cause nausea and vomiting when the drugs are used separately.^14^^,^^15^ Occurrence of these symptoms could increase after their combined use, and this may affect the acceptability of the combined treatment approach by children, their parents/caregivers, and those delivering the drugs.

Within the context of a three-arm randomized controlled trial designed to evaluate the feasibility, safety, and effectiveness of integrating helminth control with SMC (ClinicalTrials.gov NCT05354258),^16^ we have assessed the acceptability, barriers, and enablers of combining the preventive treatment with anthelminthic and SMC drugs among recipients of the drugs, their parents/caregivers, and healthcare providers (malaria and NTD program managers and health workers) in a malaria-helminth coendemic community in Senegal. The findings of this study will support the development of effective approaches to the planning and implementation of an integrated malaria-helminth control approach.

## MATERIALS AND METHODS

### Study site and context within the trial.

This qualitative study was nested within a trial conducted among preschool and school-age children in the Saraya district of Kedougou region, southeast Senegal. Saraya is ∼800 km from Dakar, the capital city of Senegal. Saraya district, which borders Mali to the east and Guinea to the south, contains 108 villages occupying a land area of 7,803 km^2^, with an estimated population of 61,756 (2019 population census). More than 70% of the population lives > 5 km from a health facility. The main ethnic groups are Malinke, Fula, Diallonke, and Diakhanke. Farming is the main occupation of Saraya residents. Malaria and helminths are highly endemic in Saraya district.^17^^–^^20^

The current trial was conducted in collaboration with the SMC and NTD programs of the Senegal Ministry of Health and Social Action. Eligible children aged 1 to 14 years were randomized into one of three groups at a ratio of 1:1:1 to determine the safety, feasibility, and tolerability of the anthelminthic and SMC drug coadministration. The first group, which served as a control arm, received vitamin A and zinc on Day 0, followed by SMC drugs on Days 1 through 3; the second group received praziquantel and vitamin A on Day 0, followed by SMC drugs on Days 1 through 3; and the third group received albendazole and praziquantel on Day 0, followed by SMC drugs on Days 1 through 3.^16^ The anthelminthic and SMC drugs are available in tablet form only. The drugs were administered to the study children according to the WHO recommended dosages, based on their ages and body weights, in the presence of their parents/caregivers. To facilitate administration of the drugs to the children, the tablets were crushed into granulated forms, and a teaspoonful of granulated table sugar was added to the crushed drugs by a trained pharmacist. All child participants were visited at home for 6 consecutive days after coadministration of the study drugs by a trained field assistant to assess the safety and tolerability of the combined drugs. Subsequently, passive surveillance was implemented to monitor any study participants who presented for admission at health facilities in Saraya. In addition, a postintervention survey was conducted 5 months after coadministration of the study medications to assess the effectiveness of the combined treatment approach. Blood, urine, and stool samples were collected from all study participants before and after coadministration of the study drugs.

### Participants and study procedures.

This qualitative study was conducted between June and December 2022 among randomly selected parents/caregivers of the child participants in the trial, using a purposive sampling method. Trial participants aged ≥ 10 years were also selected randomly for qualitative interviews. In addition, healthcare workers and trial staff who were part of the implementing team for the delivery of the study drugs were also interviewed. We also conducted in-depth interviews with managers of the SMC and NTD programs of the Senegal Ministry of Health.

### Data collection.

The data collection techniques used in this study included structured interviews, in-depth interviews, focus group discussions (FGD) and participant observations. Table 1 illustrates the profile of the study participants who were interviewed in the study. A purpose designed interview guide was used to facilitate the interviews. The interview guide explored the experiences of the parents/caregivers about the trial, their perceptions about the combined use of anthelminthic and SMC drugs, as well as other study procedures including blood, urine, and stool collection from the children, home visits for collection of safety data by trial staff, and the factors that they found encouraging or discouraging about the study. The FGD participants were separated into male and female groups to ensure homogeneity. A ring-form sitting arrangement was adopted. Expression of views was encouraged as each participant was allowed to voice their views freely and each question was discussed exhaustively until the participants raised no new topics. One-on-one interviews with study participants aged ≥ 10 years were conducted using a semistructured topic guide.

**Table 1:** Sociodemographic profile of study participants, Saraya district, 2022

Target group	Number interviewed	Gender	Age range (years)	Site, *n*		Data collection technique
Parents/caregivers	26	Male = 15 Female = 11	22–50	Saraya	4	Structured interviews and participant observations
				Khondokhu	4	
				Mandakholi	6	
				Bembou	4	
				Badioula	4	
				Dioulafoundou	4	
Study participants aged ≥ 10 years	10	Male = 6 Female = 4	10–14	Saraya Bembou Badioula Dioulafoundou		One-on-one interviews
Health care providers	4	Male = 3 Female = 1	28–40	Saraya Health center	–	In-depth interviews
Trial staff	15	Male = 13 Female = 2	18–65	Saraya	8	Focus group discussions
				Bembou	7	
Program managers	2	Male = 1 Female = 1	50–55	SMC and NTD Programs, Ministry of Health and Social Action	–	In-depth interviews

The trial staff and Saraya health facility staff were also interviewed. The health facility staff were purposively selected and included the medical director of the Saraya health center, a medical doctor at the center, a nurse, and a member of pharmacy staff. Two members of the SMC and NTD programs were also interviewed using a topic guide that included the following themes: 1) perceptions about the integrated delivery of anthelminthic and SMC drugs to children; 2) the feasibility of implementing an anthelminthic-SMC program by a joint team of SMC and NTD program officers; 3) adaptations to working practices that would be required to implement the integrated antihelminthic-SMC program, if it becomes a policy; and 4) recommendations on factors that may facilitate effective implementation of the integrated approach.

The interviews were conducted in French or in any of the local languages preferred by the study participants by the project’s social scientist (Am. D.) and notes on verbal and nonverbal communications were taken by a trained research assistant. Each interview lasted for ∼45 to 60 minutes. All interviews were digitally recorded and transcribed into English. All field notes were coded to maintain the confidentiality of participants. For quality assurance, translations and transcriptions were verified by an independent social scientist who is a native speaker of a Senegalese language and who also speaks English and French.

### Data analysis.

The first step in coding the data collected from the interviews was to transcribe the interviews into a written format. This was done manually. After this, we read through the transcripts several times to become familiar with the data and identify potential codes. We identified relevant themes by using a combination of underpinning issues in the interview guides that formed the basis of the research questions and issues that emerged during the interviews. Subsequently, we developed a codebook that outlined the codes and categories that we used to analyze the data. Two members of our team (Am. D. and Ad. D.) coded the data and used NVivo software version 12.0 (QSR International, Burlington, MA), to manage the data and apply the codes. The two researchers who coded the data met regularly to discuss their coding decisions and resolve any discrepancies. Unresolved disagreements by the two researchers were resolved after discussions with a third independent researcher (N. M. S.). As the coding process progressed, the two researchers refined the codebook and added new codes or categories, based on the emerging themes in the data. The coded data were analyzed by using the emerging themes and patterns in the data. This involved creating visualizations such as word clouds to identify similarities between common themes. We triangulated common themes arising from the interviews and included verbatim quotes of the study participants to buttress certain views expressed in the course of the interviews.

## RESULTS

Fifty-seven study participants comprising 26 parents/caregivers, 10 study children aged ≥ 10 years, 15 trial staff, four healthcare providers, and two managers from the Senegal Ministry of Health were interviewed. Thirty-eight of the participants (66.7%) were male and 19 were female (33.3%). Their ages ranged from 10 to 65 years (Table 1). The safety data obtained from the active and passive surveillance of participants after coadministration of the study drugs will be published in a separate paper. For this qualitative study, four main themes were identified from the interviews relating to the acceptability, enablers, barriers, and recommendations for implementation of an integrated anthelminthic-SMC program.

### Perception and experiences about combining drugs against malaria and helminths.

#### Perception about the combination of drugs for treatment malaria and helminths.

Most parents interviewed were happy for their children to participate in the study. They thought it was a good initiative as the diseases targeted by the study were perceived as dangerous diseases. They also reported that the occurrence of these diseases remained very high in their locality, especially in the rainy period, affecting all layers of the population, mainly children. According to some parents/caregivers, the burden of malaria and parasitic worms was due to the geography and climate of the region.

But we can only be satisfied. Because every year our children fall ill with malaria and stomach aches because of worms so if we are given medicine to treat these two diseases, we must take advantage of it. *(PC1, Dioulafoundou, parent/caregiver)*

You know we are in a border area and as each rainy season approaches, malaria and worms begin to set in. The swelling of the belly of the child are due to the worms, therefore the medicines are very important for us. *(PC2, Khondokhu, parent/caregiver)*

Comments from most parents showed that the SMC had contributed to a significant reduction in malaria burden in the locality during the past years. They also acknowledged that the risk of malaria was still present, especially during the rainy seasons when children were exposed to stagnant water where mosquitoes bred.

Children are in contact with nature eh, here they are everywhere, they wade through stagnant water during wet season. Here, at the backwater, that’s where they go to bathe or swim, so all of this can give them worm diseases. Malaria too, with the mosquitoes that develop in these places where children frequently go. So combining the drugs for malaria and worms for the children can really give a convincing result for better health of these children. *(PC4, Bembou, parent/caregiver)*.

Several parents cited the health implications resulting from combined malaria and helminths as one of the reasons for accepting that their children could participate in the study. They reported that their children suffered frequently from symptoms from fever, stomach swelling, stomach pain, or bloating. These symptoms were described in local parlance (in Mandinka language) as *halia messong*, which means “little worms.” Another term, *halia dabaa*, described as “big worms,” was said to occur when a child suffered severe growth problems. On the basis of this widely held notion, the parents were of the opinion that the combined preventive treatment of malaria and worms offered to their children, without having to travel to a health center or pay money for the drugs, was a great opportunity that they should seize to ensure that their children did not suffer from the health consequences posed by combined malaria and worm infections. Other caregivers reported that they felt honored that their children were selected among many other children in Senegal to be part of the trial. Some parents raised concerns about the continuity of the intervention and the possibility of expanding the project to reach all children in other Senegal communities.

It’s a feeling of satisfaction and a bit of worry because I’m like, “Is this going to continue?” They can get us used to this program this year, and later let us down, that’s my concern, but otherwise it’s a feeling of satisfaction and it must also be said that I felt sorry for some children to see them take many drugs at the same time, but I believed it was for their good. *(PC7, Bembou, parent/caregiver)*

Yes the feeling that I really had that moved me a lot because not only does it contribute to the good health of my children and it is a way of fighting against these diseases that we do not know, but which ravage the rural area especially in the rainy season. In any case, personally, I find this very important and it’s a feeling of satisfaction that I have because I tell myself that maybe I have good luck, I don’t know if it’s in all over Senegal. *(PC9 Dioulafoundou, parent/caregiver)*

Similar opinions were expressed by the trial staff who administered the study drugs and those who visited the children and their parents at homes for safety assessment. They considered their involvement in this project as a chance to participate in the elimination of the two major diseases (malaria and worms) that have contributed to the poor growth and death of many children in the community.

As CK said, we had a feeling of pride because before, we saw a lot of child deaths, so today it’s a pride to see them taking the drugs for their improved health from malaria and worms. *(MA1, Saraya, trial staff)*

The feeling I had was fulfillment and satisfaction because, in some way, it contributed to the development of my locality. Since these diseases can lead to several deaths, so if we participate in eradicating them, it is a source of pride for us. Seeing the children taking the medicine also reassures us that they are not going to get malaria or worms. *(MA3, Bembou, trial staff)*

However, healthcare providers expressed concerns about the possibility of increased adverse drug reactions after co-administration of anthelminthic and SMC medications. They suggested that practical strategies, such as adapting the dosages of the drugs according to age groups, should be developed to minimize the likely adverse effects of the drugs. They also said that the study plan of giving anthelminthic drugs a day before the SMC drugs might improve their tolerability.

We know that these are drugs that we are used to giving and each of these drugs cause certain side effects; so I had a little concern about the combination of these drugs and about the reaction in the children’s bodies. It was a feeling of a little worry that drove me, but still after the administration, I saw that there was really nothing to be afraid of. So, I feel a great pleasure to be part of this study. Especially since at the very beginning we were called to give an update on the situation of the malaria and worms in general, and to tell us that this year they intend to combine the drug campaigns of NTD and SMC. *(HCP 1, Saraya, health facility staff)*

My pharmacy colleagues prepared the dosages of each drug according to a child’s age or weight. It’s across age groups; for each child we administered the dose that goes with it. Like, for example, 3-year-olds and 10-year-olds cannot have the same dosage. We adapted the drugs and put them in sachets, which we gave to the medication administration team. *(HCP 4, Saraya, health facility staff)*

#### Experiences after coadministration of anthelminthic and SMC drugs.

Overall, most parents/caregivers interviewed appreciated the way the administration of the antimalarial and anthelminthic medications had taken place. The parents reported that the children took all the doses of the medications and that some children experienced some mild effects such as mild fever, diarrhea, vomiting, and drowsiness. The parents stated that the information provided by the study team during community sensitizations allayed their fears about the combined medications, and any possible side effects. According to them, in most cases, the side effects did not last more than a day.

I am happy, I also had strong confidence that my child will be protected from these diseases. But there were side effects as the child slept all day after taking the medicine, he also had mild fever but these side effects don’t last long, and that’s normal too.

How long did they last? *(Investigator)*

Only 24 hours, now he is fine.

Why do you say side effects are normal? *(Investigator)*

It’s normal because the drugs are not the same if each one does its job, the child must feel it in his body. *(PC3, Bembou, parent/caregiver)*

Yes, I noticed that one of my children had mild diarrhea, otherwise they remain calm all day, it even looks like they are sick. But the side effects did not last long, now they are doing very well. *(PC5, Dioulafoundou, parent/caregiver)*

And they gave out a health book telling us that if our kids get sick after the child took the medications, you take them to the health center with the book. They told us that if you go to the hospital, you’re not going to pay anything for the drugs, it’s them who will pay for the drugs. *(PC6, Badioula, parent/caregiver)*

There was consistency in the views expressed by the parents, healthcare providers, and trial staff that the refreshments (snacks and glucose drinks) provided to the children before administering the medications were effective tools because they helped facilitate children’s uptake of the drugs. Some caregivers noted that children who usually disliked taking drugs quickly cooperated when they saw the snacks. The trial staff who administered the drugs reported that the first day, when they distributed the drinks and snacks, was the day when their work was in administering the drugs was the least challenging.

It’s really the biscuits and drinks that made the job easier and even the children who didn’t take the medication asked their parents why they didn’t receive it like their siblings who took it. That’s really what made the job easier. *(PC8, Khondokhou, parent/caregiver)*

I will rather talk about what made the job easier. That is, the cookies on the days that we took with us, it made the job a lot easier. When the children saw that their siblings were taking the medicine and then received biscuits, they too came to take it”. *(MA5, Saraya, trial staff)*

Some healthcare providers emphasized that the initial objective of giving out snacks to the children was to prevent them from taking the drugs on an empty stomach, which could accentuate the side effects. However, the healthcare providers realized that the snack distribution was a major factor that facilitated the acceptability of the drugs by many children.

The goal was for the children to be able to have something in the stomach before taking the medicine. But we found it to be a motivator for some who saw these snacks even though it was not included in the study, there were children who wanted to be given the drug just to get the snacks and drinks. *(HCP2, Saraya, health facility staff)*

A small number of parents complained that the size and bitter taste of some of the drugs made many children reluctant to take them. To overcome this challenge, they reported that crushing the drugs in mortars and adding sugar to them, as done in the study, reduced the pill burden and made the drugs easier for the children to swallow.

As soon as the health workers came to our household, I gathered the children concerned and I saw the health workers were grinding the drugs in a small mortal and giving them to the children. This made it easier for the children to take medicine in addition to the snacks given to the children, this motivates the children to take because they have taken the juices and the snacks. *(PC10, Dioulafoundou, parent/caregiver)*

### Enablers of acceptability of combined medications.

Having established the acceptability of the integrated malaria–helminth treatment approach by the users, we explored further factors that might have contributed to the positive feedback. These included the following.

#### Perceived effectiveness of SMC.

Interviews with the parents/caregivers of the children who received the combined drugs revealed that the perceived effectiveness of SMC was the primary factor that motivated their willingness to enroll their children in the trial. Most of the parents interviewed emphasized the decline in malaria burden among Saraya children was due to the annual implementation of SMC campaigns. They maintained that the rainy season was usually a difficult time for children in the community because of the increased incidence of malaria and the associated complications. They observed that most children who took the SMC drugs during the campaigns did not develop malaria throughout the rainy season. Many parents and trial staff narrated specific cases where children died after parents’ refusal to allow their children to take SMC drugs.

I witnessed a fact in my household, my brother refused to administer the malaria drugs to his children. I didn’t know initially that he did this because I was in another area. A few days later, one of his daughters fell ill with malaria, we took her to the health center and was referred to Kédougou regional hospital but she eventually died. That’s why I say the district needs to take action on parents who withhold drugs from their children. *(MA7, Bembou, trial staff)*

But every year we do prevention against malaria and the children who take their malaria medicine regularly have not had the difficulty of contracting malaria during the wet season, as we understand the difference between the children who have taken the medicine and the children who did not take the drug. It is useful for children because the difference is that these children, as I said, do not have malaria unlike the others who had not taken it. *(PC10, Mandakholi, parent/caregiver)*

Given the aforementioned demonstration of confidence in the benefits of SMC, many parents/caregivers expressed their belief that the combined malaria–helminth treatment approach would have the same results in rolling back malaria and helminths.

Because we understood the usefulness of SMC drugs in successfully rolling back malaria. We understood, we saw the results. The same thing will happen for this new approach. *(PC13, Dioulafoundou, parent/caregiver)*

I accepted because I saw the benefits of the SMC malaria campaign. I agreed because I believe the benefits of this combined treatment will be successful like that of SMC, and I don’t want my kids to get sick again because of malaria and worms. *(PC16, Badioula, parent/caregiver)*

#### Trust in government health programs.

Some parents also explained that their acceptance of the combined drugs was based on their trust and confidence in the health programs sponsored by the government. For them, the government would not implement any programs that could endanger the lives of their children. They considered that decisions taken by government health authorities were safe and reliable, so they would not hesitate to accept them.

You know, the government and the population is like the father of a family and his children. A father never endangers his own children, he protects them. So, I believe that if the government gives drugs to the population, it is for their good. The government cannot move health workers and investigators from Dakar to Kédougou to give the wrong drugs to the population. In addition, each year, the government gives us treated mosquito bednets to fight against malaria, so if they give us this medicine, I think it is to reinforce the prevention measures. *(PC14, Dioulafoundou, parent/caregiver)*

If it was another project different from the government project, I would be afraid and worried about the things they give to our children, but as it is the government which is at the origin of this project so that our children can be prevented from malaria and worms, so I will make sure to reciprocate their kind gesture. *(PC17, Saraya, parent/caregiver)*

#### Perception on feasibility of the drug combination.

In the opinion of NTD and SMC program managers, the initiative to combine preventive drugs against malaria and helminths is a timely approach in the context of the scarcity of economic and human resources prevailing in Senegal. The managers highlighted the relevance of the integrated treatment approach in reducing the public expenditures for implementing separate vertical mono-disease control programs. They also emphasized that the combined approach would enable them to gain time that could be channeled into developing operational planning and strategic implementation of other activities at the Ministry.

The idea of combining campaigns for the same target population seems to me to be very efficient, in order not only to save financial resources but also resources in terms of time for the organization of these campaigns and which nevertheless allows better integration of activities at the level of the Ministry of Health. *(PM1, program manager)*

What we find interesting in this combination is the fact of maximizing synergies, especially since we are working on the same target population, and we are the same actors, operating in the same health system. So, it’s important that we are moving towards this integration as it will allow us to be much more time-efficient, ensure better cost-efficiency in the delivery of the intervention especially at the operational level. It will also reduce the workload of the service providers. *(PM2, program manager)*

Similar comments were made by the healthcare providers who reported that community fatigue usually sets in when multiple MDA campaigns are implemented in the same communities within a short timeframe.

Apart from these campaigns, there are other campaigns that are taking place throughout the year; an example is the expanded immunization program, there are so many other campaigns that are carried out, so if we reduce the number of campaigns by integrating them, that would certainly allow for better acceptability. *(HCP4, Saraya, health facility staff)*

In order not to repeat myself, I can say that it is the fact of combining the drugs. At the strategic level, having better efficiency is beneficial. The two programs are in the same direction, and we do not have the resources for several campaigns. Especially since the campaigns are done with the same actors with the same target populations. *(PM2, program manager)*.

### Barriers to acceptability of combined treatment approach.

The parents/caregivers, trial staff, and healthcare providers provided insights on the challenges they encountered when delivering the integrated anthelminthic-SMC drugs to the children. These perspectives are highlighted under the following headings.

#### Reluctance/refusal by children.

Refusal to take the combined drugs was reported by some children across all age groups but was most pronounced among 10 to 14-year-old children. It was difficult to find these children at home at the time of the drug administration, and some deliberately left home on drug administration days and did not return home until late in the night. The trial staff felt this behavior was probably because this was the first time that the adolescents were included in the SMC campaigns. This notion was corroborated by a group of adolescents who were interviewed to understand why they refused to take the medications. Some admitted that that they were ashamed of being asked to urinate or pass stool in a sample collecting bottle, especially by a trial staff member of the opposite sex. Others attributed their reluctance to the mockery they suffered from their friends who said they were treated as younger children whose stool and urine were collected after receiving snacks.

We encountered a lot of difficulty, especially when we went to a house and we can’t find the child concerned, in which case we have to leave and come back because we don’t leave the medicines with the mothers, because it is not sure that they will administer the drugs to the children, which is why sometimes we stay in the field until 11 pm. *(MA13, Bembou, trial staff)*

The conversations below between the interviewer and a parent also underscored the gender sensitivity displayed by the adolescent participants:

I remember that, because I had a child, a boy who refused to allow these people to take his stool.

How old was the child? *(Investigator)*

He is 13 years old today, I believe.

Why do you think he refused? *(Investigator)*

The first day they came to my house to take his stools, at the time I had gone to the farm. But when I arrived, his mother told me that. I asked him why he refused, and he told me that it was a woman who had wanted to take his stool. So I asked the staff to come back because they were still here in the village and I personally took the stool and I gave it to a gentleman.

So, if I understand correctly, it’s the fact that it’s a woman that the boy refused?” *(Investigator)*

For the boys it must be men and for the women too because they are ashamed to see a woman take their stool like that, it bothers them. *(PC22, Khondokhu, parent/caregiver)*

Refusal of the medications was also predicated on the size and taste of some of the drugs. The trial staff and healthcare providers submitted that albendazole had a pleasant taste, which made it more acceptable to the children while amodiaquine and praziquantel are bitter and consequently, many children were reluctant to take them, and some rejected them totally.

The first day that we started giving the drugs to the children, we had problems with them, because the drugs are bitter. Some take it straight, but for others you have to pound the medicine to give it to them. The drug like praziquantel is a bit big. Now I don’t know what to do, but I think we will have to try to see another alternative, it can be the same tablet, but it will have to be reduced in size. And also, if it’s possible to do it with a little sugar, so swallowing it will be much easier. *(MA6, Badioula, trial staff)*

### Recommendations.

#### Parents/caregivers.

The recommendations provided by most parents centered on the bitter taste of some of the drugs. Some parents felt that preparing the tablets in granular form and adding granulated sugar did not remove their unpleasant smell and taste. They agreed unanimously that the smell and taste were the causes of nausea and vomiting observed in the children, which made other children refuse to take the drugs. The parents also recommended minimizing side effects from taking medication as much as possible.

The problem with the medicine is the bitter taste. This is what scares the children. We would have preferred it to be easy to take like paracetamol. Because even this 4-year-old child who is there, when I give him the paracetamol, he takes it without difficulty. So, I would have liked that the drugs we give to the children didn’t have a bitter taste. *(PC24, Badioula, parent/caregiver)*

If they really can change the taste and also the smell, that would be good; because the taste and smell of some of the drugs are problems to the children. That’s what makes children vomit. *(PC21, Dioulafoundou, parent/caregiver)*

#### Trial staff, healthcare providers, and program managers.

Similar recommendations to those of the parents/caregivers were made by the trial staff, healthcare providers and program managers.

The recommendation is to improve the taste of the drugs as well as the smell if possible so that these drugs will be much easier to take and absorb. *(MA8, Saraya, trial staff)*

It is important during the organization of these campaigns to make available to health structures the necessary means to take charge correctly, and in communication to inform the populations, because the principle of medicine is to do no harm: if we give drugs that are not, and which have side effects, we must not make the parents bear the burden and therefore, we must take steps so that the local health authorities, that whether regional, whether hospital, district or health post level, can have the means to take charge of these cases in case side effects may occur. *(PM1, program manager)*

They also advocated using appropriate awareness-raising strategies that take into consideration the nuances of the adolescent population. Collaboration with the education sector through the school-based health platform was cited as an appropriate channel to better reach the adolescents who are school going. Using social media platforms to deliver strategic messages that could cater for the information needs of the adolescents was also identified as a way to strengthen the awareness program on the integrated anthelminthic–SMC approach.

In any campaign there are refusals that are always recorded; the solution is to organize during these campaigns management committees for cases of refusal to do the advocacy, and also to guarantee to these populations the safety of what we are doing and once they are convinced, I believe they will be able to adopt and agree to take these drugs. *(HCP3, Saraya, healthcare provider)*

Use all possible channels, currently social networks are very popular with this age group, we have TikTok, we have Facebook, we have all these current tools. I think we will have to adapt the communication to this population through social media and similar networks because young people no longer listen to the radio, or watch local TV stations like before. We can also reach them through their associations, through WhatsApp groups and others to ensure that there is a clear message that can be digested by these young people. *(PM2, program manager)*.

Concerning the possibility of establishing a joint team of SMC and NTD program officers to adapt working practices and implement an integrated treatment approach, program managers were unequivocal in their support to work towards entrenching such partnerships. They reiterated the benefits inherent in integrating the control programs to minimize cost and maximize efficiency in delivery and health outcomes of the target population.

Everything will depend on the results of this study and the different aspects that I have talked about because the overall advantage of this strategy is to save time and money to organize the two separate campaigns because the Ministry of Health is used to organizing these two campaigns. If the results of the final evaluation shows that there is efficacy and safety in the administration of these combined drugs, why would we not make recommendations in this direction? *(PM2, program manager)*

First you have to disseminate the findings of this study because all things you have gathered in this study needs to be shared with all the players. The different actors need to discuss together to find a solution, and move towards this combination of treatment, especially in areas with high prevalence. More importantly, we know that we have to carry out several interventions, and we are all convinced of the benefits of integration. We have even set up a pilot project to integrate NTD with the other existing platforms in the district of Koungheul. So to ensure the sustainability of our interventions, we have to move towards integration. *(PM1, program manager)*

## DISCUSSION

Senegal has been implementing SMC and MDA for STH and schistosomiasis as separate vertical mono-disease control programs since 2008 and 2009, respectively. Seasonal Malaria Chemoprevention was first evaluated in Senegal in 2008 before its eventual adoption by the WHO for implementation across the Sahel region in 2012.^21^ The sustained implementation of these two programs has contributed to the reduction of the burden of malaria and STH in Senegal and moved the country to the pre-elimination stage for the two diseases.^22^^,^^23^ In line with the global agenda of eliminating malaria and some NTDs by 2030,^24^ we conducted this study to assess the provider and user acceptability of an integrated control treatment of malaria and helminths.

Our findings showed that coadministration of anthelminthic and SMC drugs was acceptable to the children, their parents/caregivers, and the implementing team. These findings are consistent with the results of similar studies that have assessed the acceptability of antimalarial^25^^,^^26^ or anthelminthic medications.^27^^,^^28^ Although there were concerns about the unpleasant taste of two key medications (amodiaquine for SMC and praziquantel for schistosomiasis), this did not have a negative impact on the overall acceptance of the integrated treatment approach. This may be due in part to the pragmatic approach adopted in this study to transform the drugs into granulated form by trained pharmacists. The addition of granulated table sugar to the granulated medicines also improved the taste. Sweetening agents such as sucrose are frequently added to pharmaceutical dosage formulations to mask the bitter taste of partially dissolved drugs and to improve palatability in general.^29^ This method was adopted because of the relative availability of table sugar in the resource-limited setting where the study was conducted. It is hoped that the manufacture of flavored pediatric formulations of amodiaquine and praziquantel will address this concern.

All study participants raised concerns about the side effects associated with the bitter taste of amodiaquine and praziquantel, which led to severe nausea and vomiting in a minority of children. Previous studies have documented the increased incidence of these adverse events with amodiaquine^15^^,^^30^ and praziquantel.^31^^,^^32^ Accurate dosing of age-appropriate, pediatric-friendly formulations is needed to reduce the incidence of these adverse events and improve the palatability of the drugs.^31^^,^^33^

Trust and confidence in government-sponsored health programs came out strongly as the major factor that influenced the acceptability of the integrated malaria–helminth preventive approach deployed in this study. A high level of trust in government and health institutions has been reported in many studies to correlate with high uptake of interventions and improved health-seeking behaviors.^34^^,^^35^ Nevertheless, findings from our studies showed that many of the parents/caregivers believed that the study was specifically developed for the care of their children, a concept described as “therapeutic misconception.”^36^^,^^37^ This might be due to the involvement of government staff who were known in the community to be responsible for the implementation of SMC and MDA for STH and schistosomiasis. This study was developed and implemented in collaboration with the Ministry of Health, and this strengthened its acceptance within the study community. However, when similar collaborative studies between researchers and the Ministry of Health are undertaken in the future, it is important that the expectations of the study population are not raised inappropriately by stressing that the intervention is only at an experimental stage.^38^ Similarly, snacks were provided to the children as a part of the research implementation to ensure that the study medications were not taken on empty stomach because this could exacerbate the drug–drug interactions between praziquantel and amodiaquine.^39^^,^^40^ Although snacks are not routinely provided in real-life situation during programmatic implementation, children are usually encouraged to eat before taking the medications. This may affect acceptability, uptake, and scalability of the integrated treatment approach.

Refusal and/or rejection of the combined drugs was reported among children who were in the 10- to 14-year age group. On exploration of the factors driving this behavior, it was discovered that peer influence and lack of sensitivity to adolescent sexuality played a significant role. Studies have reported similar findings on how normative beliefs and the behavioral influence of the interactions among peers have hindered young people from utilizing health services because of the fear of stigmatization and gossip.^41^^,^^42^ To overcome this challenge, culturally appropriate and gender-sensitive approaches should be adopted for the administration of the medications and collection of biological samples such as urine or stool from adolescent participants. Also, given their unique physiological changes, specific barriers affecting the adolescents’ acceptability and uptake of the integrated treatment approach should be addressed by involving the adolescents in co-creation and implementation of tailored health and education services that reflect their developmental stage and unmet needs.^43^

The program managers and healthcare workers provided encouraging insights on the feasibility of a harmonious integration of SMC and MDA for STH and schistosomiasis control. This submission was probably driven by the perceived benefits of cost-cutting and improved efficiency in operational planning and implementation of an integrated control program for malaria and helminths. Although these efforts will involve national and international harmonization and coordination of the activities of partnerships devoted to control or elimination of the two major diseases,^44^ it is imperative to embrace the increasingly recognized strategy of integration of SMC and MDA for STH and schistosomiasis control into a broader health systems to optimize system effectiveness and cost-effectiveness, particularly in health systems with limited human resources, as frequently found in SSA. The integration also has the potential to accelerate progress toward universal health coverage and advancing the 2030 agenda.^45^

Our study had a few limitations. The opinions and views expressed by participants in this study were shaped largely by their sentiments on the effectiveness of SMC program in the community and in Senegal as a whole. A key challenge arose in separating the participants’ opinions about the integrated anthelminthic–SMC approach from the component of the SMC intervention because the two were closely linked in this study. Given that the study participants had not experienced an integrated malaria–helminth treatment approach before this study implementation, it was also difficult to clearly separate their views on the integrated approach from similar MDA campaigns that took place previously in the community. Also, a preponderance of male participants in this study might have reduced the information on the lived experiences of mothers who usually played the socially ascribed roles of primary caregivers in most African communities. Nevertheless, our approach of using different interviewing techniques helped to minimize the response bias that might have undermined the findings of this study.

In conclusion, we report the findings of the first study that has explored the providers and caregivers’ acceptability of an integrated malaria–helminth treatment approach within the context of a clinical trial in Senegal. Overall, the approach was well accepted by the study participants, but concerns about the taste and smell of amodiaquine and praziquantel need to be addressed to optimize the uptake of the integrated control program. Also, culturally appropriate strategies need to be put in place to cater for the inclusion of children aged 10 to 14 years if the integrated malaria–helminth control program is to become an international policy.
